# Identification of novel targets associated with cholesterol metabolism in nonalcoholic fatty liver disease: a comprehensive study using Mendelian randomization combined with transcriptome analysis

**DOI:** 10.3389/fgene.2024.1464865

**Published:** 2024-09-18

**Authors:** Juan Chen, Huajing Rao, Xiaoling Zheng

**Affiliations:** ^1^ Department of Gastroenterology, Shengli Clinical Medical College of Fujian Medical University, Fujian Provincial Hospital, Fuzhou University Affiliated Provincial Hospital, Fuzhou, Fujian, China; ^2^ Emergency Internal Medicine, Affiliated Fuzhou First Hospital of Fujian Medical University, Fuzhou, Fujian, China; ^3^ Department of Endoscopy, Shengli Clinical Medical College of Fujian Medical University, Fujian Provincial Hospital, Fuzhou University Affiliated Provincial Hospital, Fuzhou, Fujian, China

**Keywords:** NAFLD, cholesterol metabolism, prognosis, bioinformatic analysis, Mendelian randomization

## Abstract

**Background:**

There is limited research on cholesterol metabolism-related genes (CM-RGs) in non-alcoholic fatty liver disease (NAFLD), despite hypercholesterolemia being a recognized risk factor. The role of CM-RGs in NAFLD remains unclear.

**Methods:**

The differentially expressed genes (DEGs) between NAFLD and control were acquired by differential expression analysis. The differentially expressed genes associated with cholesterol metabolism (DE-CM-RGs) were identified and functional enrichment analyses were performed. Protein-protein interaction network analysis and a two-sample Mendelian randomization study were utilized for identifying hub genes. Nomogram model, competing endogenous RNA and messenger RNA-drug networks were established. In addition, immunoinfiltration analysis was performed.

**Results:**

We identified four hub genes (MVK, HMGCS1, TM7SF2, and FDPS) linked to NAFLD risk. MVK and TM7SF2 were protective factors, HMGCS1 and FDPS were risk factors for NAFLD. The area under the curve values of nomograms in GSE135251 and GSE126848 were 0.79 and 0.848, respectively. The gene set enrichment analysis indicated that hub genes participated in calcium signaling pathways and biosynthesis of unsaturated fatty acids. NAFLD patients showed increased CD56^dim^ NK cells and Th17. Tretinoin, alendronate, zoledronic acid, and quercetin are potential target agents in NAFLD.

**Conclusion:**

Our study has linked cholesterol metabolism genes (MVK, HMGCS1, TM7SF2, and FDPS) to NAFLD, providing a promising diagnostic framework, identifying treatment targets, and offering novel perspectives into its mechanisms.

## 1 Introduction

Non-alcoholic fatty liver disease (NAFLD) is the prevailing type of chronic hepatic disorders globally. The incidence of NAFLD is continuously increasing, leading to higher mortality rates and financial burden. The analysis from 1990 to 2019 found that NAFLD prevalence globally was 30.1% in 2023 (Younossi et al., 2023), higher than the reported rate of 25% in 2016 (Younossi et al., 2016). Prompt identification and understanding of its pathogenesis are crucial to prevent irreversible damage, as there is currently a shortage of approved pharmaceutical interventions for managing NAFLD.

Metabolic syndrome, obesity, insulin resistance, type 2 diabetes mellitus, and dyslipidemia are established risk factors for hepatic steatosis (Rinella et al., 2023). The precise etiology of NAFLD remains elusive. The current “multiple parallel hits” theory mechanism mainly includes dyslipidemia, mitochondrial oxidative damage, endoplasmic reticulum stress, genetic differences, changes in immune response and intestinal microbiota imbalance (Rinella et al., 2023). Lipotoxicity refers to the disruption of lipid homeostasis and/or alteration in intracellular lipid composition, resulting in the accumulation of deleterious lipids that may potentially contribute to cellular dysfunction, injury, or demise of cells and organelles. NAFLD is characterized by the accumulation of triglyceride-containing lipid droplets in hepatocytes, which are currently considered as a defensive response to lipotoxicity (Papazyan et al., 2016). The accumulation of lipotoxic lipids, such as cholesterol, free fatty acids, and ceramides, is believed to cause cellular dysfunction and contribute to NAFLD progression (Marra and Svegliati-Baroni, 2018).

Cholesterol metabolism is crucial for maintaining liver health as it impacts the structural integrity and fluidity of cell membranes, and participates in vital biochemical processes. In NAFLD, there is a pervasive dysregulation of cholesterol homeostasis, leading to augmented cholesterol synthesis and uptake, as well as impaired clearance, resulting in elevated hepatic cholesterol levels. Hepatic accumulation of cholesterol can lead to the development of steatosis, oxidative stress, and inflammatory responses (Ioannou, 2016), thereby promoting the advancement of NAFLD. Long-term consumption of a high-cholesterol diet in mice has been linked to hepatocellular carcinoma (HCC) development, possibly due to gut microbiota dysbiosis and reactive oxygen species accumulation caused by a high-fat diet (Zhang et al., 2021).

The application of bioinformatics approaches has facilitated the discovery of biomarkers and possible targets for various diseases. In this study, we leveraged NAFLD-related public datasets from the Gene Expression Omnibus (GEO) database to identify differentially expressed genes (DEGs) through differential expression analysis. The differentially expressed genes related to cholesterol metabolism (DE-CM-RGs) were identified, protein-protein interaction (PPI) networks were constructed to identify important gene clusters and modules. The study used two samples to identify hub genes through Mendelian randomization (MR) analysis. The impact of hub genes on clinical diagnosis was assessed using receiver operating characteristic (ROC) curve and nomogram, while the correlation between hub genes and immune cells was investigated through single-set gene set enrichment analysis (ssGSEA). The primary aim of this investigation was to uncover potential DE-CM-RGs that hold diagnostic significance for individuals with NAFLD. Additionally, we established competing endogenous RNA networks and messenger RNA (mRNA)-drug regulatory network, thereby presenting a potential therapeutic strategy for the management of NAFLD.

## 2 Materials and Methods

### 2.1 Data download

The GSE135251 and GSE126848 datasets were acquired from the GEO database, containing clinical features and gene expression data from liver tissue of NAFLD patients and healthy controls. The GSE135251 dataset included ten control cases and 206 NAFLD patients, while GSE126848 consisted of 14 control cases and 31 NAFLD patients. Detailed information on the datasets was provided in Supplementary Table S1. A total of 140 cholesterol metabolism-related genes (CM-RGs) were obtained from the Molecular Signatures Database (MSigDB, https://www.gsea-msigdb.org/gsea/msigdb/index.jsp) (Tang et al., 2022). Genome-wide association study (GWAS) summary data for NAFLD was available at https://gwas.mrcieu.ac.uk/datasets/finn-b-NAFLD/, generating 16,380,466 single nucleotide polymorphisms (SNPs) from 894 European patients with NAFLD and 217,898 healthy European controls. Peripheral blood expression quantitative trait loci (eQTL) GWAS data for exposure factors were obtained from the IEU-OPCOSnGWAS database, and eQTL summary level statistics were obtained from the cap analysis of gene expression (CAGE) study, which included peripheral blood gene expression data in 2,765 individuals (Lloyd-Jones et al., 2017).

### 2.2 Differential expression analysis

The DEGs were identified using “DESeq2” R package (Love et al., 2014) with the threshold of |log_2_FC| > 0.5 and adjusted *p* < 0.05 in the NAFLD and healthy controls of GSE135251 and GSE126848 datasets. Subsequently, volcano plots and heatmaps were constructed to visually represent the DEGs in NAFLD. The “VennDiagram” package was used to obtain DE-CM-RGs by intersecting DEGs with CM-RGs.

### 2.3 Functional enrichment analysis, correlation analysis, and PPI network of DE-CM-RGs

Chromosome location information is essential for the precise positioning of genes in the genome and their upstream and downstream regions, which is beneficial for the in-depth understanding of gene functions and their regulatory mechanisms. The location of DE-CM-RGs on chromosomes was visualised using the “OmicCircos” package (Hu et al., 2014) in this study. To explore the role of DE-CM-RGs in specific biological processes and changes in the activity of specific signalling pathways, Gene Ontology (GO) and Kyoto Encyclopedia of Genes and Genomes (KEGG) enrichment analyses for DE-CM-RGs were conducted using the “clusterProfiler” package (Wu et al., 2021) in R software (adjusted *p* < 0.05). Subsequently, Spearman correlation analysis of DE-CM-RGs in GSE135251 was performed using the “corrplot” package with a 95% confidence interval (CI). The PPI network of 20 DE-CM-RGs was developed using the Search Tool for the Retrieval of Interacting Genes database (STRING, http://string-db.org) with a medium confidence level above 0.4 and visualized in Cytoscape V3.8.2 software after removing isolated targets. The molecular complex detection (MCODE) plugin integrated within Cytoscape was employed to detect Cluster1 gene based on filter criteria including a minimum degree threshold of 2, a node score threshold of 0.2, a k-core value set at 2, and a maximum depth limited to 100. These module genes were deemed as potential hub genes.

### 2.4 Mendelian randomization (MR)

In the current study, two-sample MR analysis was used to explore the causal association between potential hub genes and NAFLD. We used potential hub genes (Supplementary Table S2) as exposure variables and NAFLD as the outcome measure. The “TwoSampleMR” package (Hemani et al., 2018) was used for the MR analysis. The instrumental variables (IVs) selected for MR analysis must meet three key assumptions: Assumption 1, there should be a strong correlation between the IVs and exposure factors; Assumption 2, the IVs are independent of confounding variables that may affect both exposure factors and the outcomes; Assumption 3, the affect of the IVs on the outcomes is solely mediated by their influence on the exposure factors, excluding any other mechanisms. SNPs related to hub genes and were not linked to NAFLD were selected by reading exposure factors and filtering IVs through the “extract_instruments” function in R package TwoSampleMR with *p* < 5 × 10^−8^. The SNPs in linkage disequilibrium were eliminated with clump = TRUE, *r* ^2^ = 0.001, and kb = 20. kb = 20 was used to increase the number of SNPs available for analysis. MR analysis was performed using five algorithms, including MR Egger (Bowden et al., 2015), weighted median (Bowden et al., 2016), simple mode (Hemani et al., 2018), inverse variance weighted (IVW) method (Burgess et al., 2015), and weighted mode (Hartwig et al., 2017). The impact estimates were mainly computed using the IVW method. According to the IVW method, if the corresponding *p*-values of hub genes were less than 0.05 and their odds ratio (OR) was greater than 1, these genes would be considered risk factors for NAFLD, and if the OR values were less than 1, they would be considered protective factors for NAFLD. Hub genes with *p*-values greater than 0.05 were considered to have no causal association with NAFLD. As the MR results, they were presented using scatter plots, forest plots, and funnel plots.

### 2.5 Sensitivity analyses

Sensitivity analyses, comprising heterogeneity test, horizontal pleiotropy test, and leave-one-out (LOO) analysis, were conducted to assess the validity and generalisability of the MR findings. We detected heterogeneity among the causal effects through the mr_heterogeneity function, using the Cochran’s Q statistic from the IVW methodology. A *p*-value of greater than 0.05 for the Cochran’s Q test indicated the presence of heterogeneity (Xu et al., 2023). Horizontal heterogeneity test was supported by the “mr_pleiotropy_test” function, respectively (Qin et al., 2023). The *p*-value of MR-Egger exceeded 0.05 manifested the absence of horizontal pleiotropy in MR study. We utilized “mr_leaveoneout” function for LOO analysis (Cui et al., 2021), which evaluated the effect of the remaining SNPs on the outcome variable by progressively eliminating each SNP through IVW method.

### 2.6 Evaluating the diagnostic accuracy of hub genes

The “pROC” package (Robin et al., 2011) was used to evaluate ROC on datasets GSE135251 and GSE126848. The genes used for diagnosis have an area under the curve (AUC) value of 0.7 or higher. The accuracy of a diagnostic nomogram, constructed based on gene markers, was evaluated using a calibration curve.

### 2.7 Gene set enrichment analysis (GSEA)

The “clusterProfiler” package (Yu et al., 2012) was used for GSEA, which identified significant functional and pathway differences among groups with varying expression levels of hub genes in the GSE135251 dataset. We selected the reference KEGG gene set and GO gene sets from the MSigDB. The significance level for GSEA was set at a threshold of less than 0.05, considering the adjusted *p*-value.

### 2.8 Immune analysis

The ssGSEA algorithm from the “GSVA” package (Hänzelmann et al., 2013) was used to evaluate the infiltration of 28 immune cells in the GSE135251 dataset between the NAFLD and control groups. Heat maps were generated to show differences in immune infiltration. The Wilcoxon test was employed to evaluate the disparity in immune infiltrating cells between NAFLD and control groups (*p* < 0.05). A boxplot was used to display the differential immune infiltrating cells. The “corrplot” package was used to analyze the correlations between differential immune cells. The Spearman correlation analysis was utilized to identify significant correlations and a scatter plot was created to visually represent the relationships between hub genes and immune cells.

### 2.9 Construction of regulatory networks

According to the NetworkAnalyst platform (https://www.networkanalyst.ca/), the encyclopedia of DNA elements (ENCODE) database was utilized for the prediction of transcription factors (TFs) linked to hub genes. The TarBase and miRTarBase databases were utilized to predict the microRNA (miRNA)-mRNA interactions based on these hub genes. Additionally, evidence for direct interaction between the predicted miRNAs and long non-coding RNAs (lncRNAs) was integrated using the StarBase database (http://starbase.sysu.edu.cn/starbase2/index.php). A comprehensive network comprising mRNA, miRNA and lncRNA was constructed and represented using Cytoscape software (version 3.8.2). The Comparative Toxicogenomics Database (https://ctdbase.org/) was employed to predict drugs targeting hub genes. The investigation examined the correlation between hub genes and drugs, only retaining drugs with a “Reference Count” greater than 1. The mRNA-drug regulatory network was visualized using the Cytoscape software.

### 2.10 Statistical analysis

Statistical analysis was conducted utilizing R software (version 4.1.0). A level of significance lower than 0.05 indicated the presence of a significant difference. The Wilcoxon test was employed to compare groups.

## 3 Results

### 3.1 Totally 20 DE-CM-RGs and 9 potential central genes were identified

**FIGURE 1:**
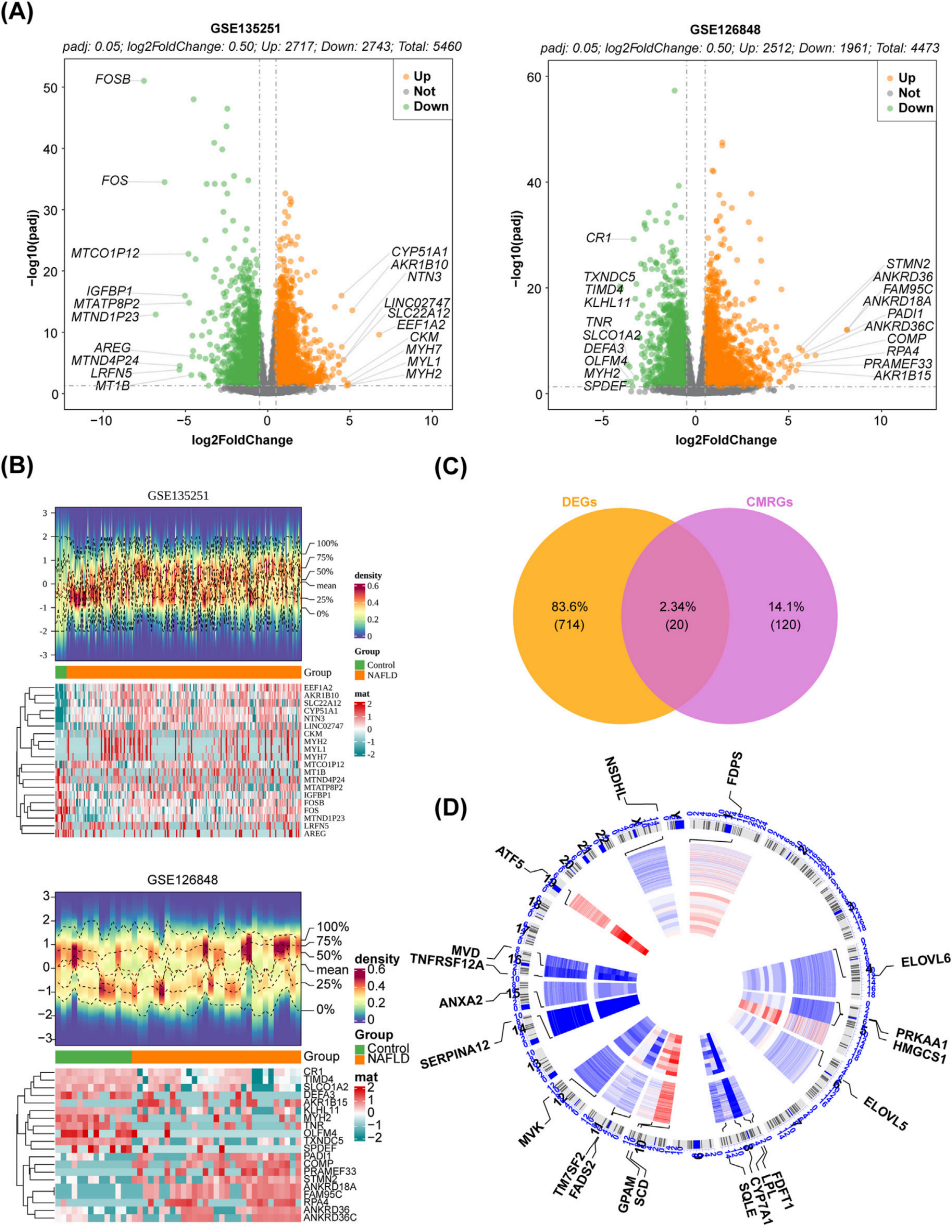
Identification of DE-CM-RGs. **(A)** Volcano plot of DEGs between the NAFLD and control groups in two GEO datasets (*p* < 0.05). **(B)** Heatmap of DEGs between the NAFLD and control groups (*p* < 0.05). **(C)** The venn diagram illustrating the intersection of DEGs and CM-RGs. **(D)** The locations of the 20 DE-CM-RGs on 23 chromosomes. CM-RGs, cholesterol metabolism-related genes; DE-CM-RGs, the differentially expressed genes associated with cholesterol metabolism; DEGs, the differentially expressed genes; GEO, Gene Expression Omnibus.

**FIGURE 2:**
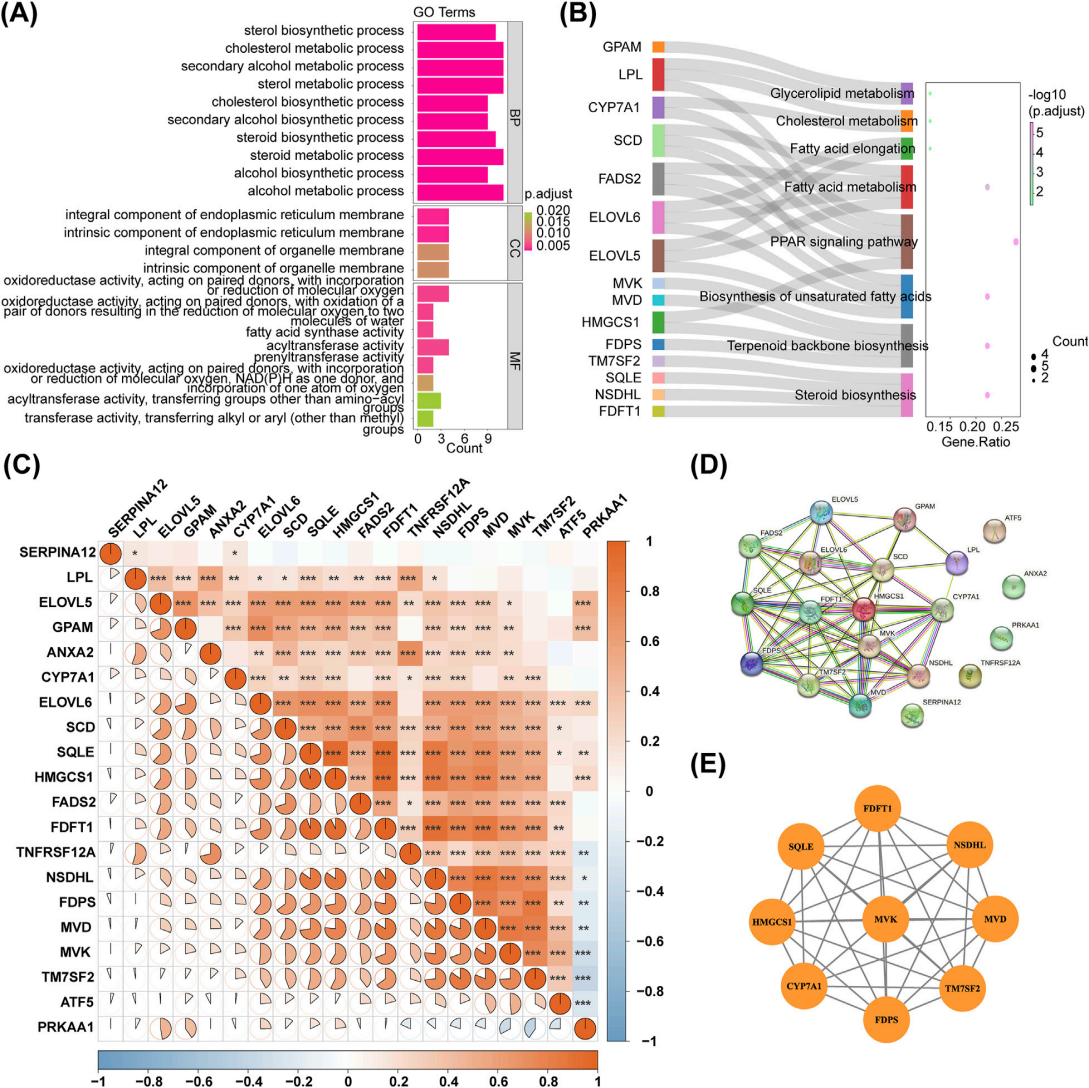
PPI network construction for DE-CM-RGs. **(A,B)** GO terms and KEGG pathways enriched by DE-CM-RGs. **(C)** Correlation analysis of 20 DE-CM-RGs. **(D)** The PPI network of DE-CM-RGs. **(E)** The most significant module identified by MCODE. PPI, protein-protein interaction; DE-CM-RGs, the differentially expressed genes associated with cholesterol metabolism; GO, Gene ontology; KEGG, Kyoto Encyclopedia of Genes and Genomes; BP, biological progress; CC, cellular component; MF, molecular function; MCODE, molecular complex detection.

In the GSE135251 dataset, totally 5,460 DEGs were obtained between NAFLD patients and healthy controls, with 2,717 upregulated genes and 2,743 downregulated genes. The dataset GSE126848 identified 4,473 DEGs, with 2,512 upregulated genes and 1,961 downregulated genes. The volcano plot and heatmap of the two databases were shown in Figures 1A,B. The up- or downregulated DEGs in GSE135251 and GSE126848 were intersected separately, resulting in 455 upregulated and 279 downregulated genes. Taking the intersection of the upregulated DEGs (2,717) in GSE135251 and the upregulated DEGs (2,743) in GSE126848, 455 shared upregulated DEGs were obtained. Taking the intersection of the downregulated DEGs (2,512) in GSE135251 and the downregulated DEGs (1961) in GSE126848, 279 shared downregulated DEGs were obtained. Shared upregulated DEGs and shared downregulated DEGs were combined to obtain 734 shared DEGs. Twenty DE-CM-RGs were identified by crossing 140 CM-RGs with 734 DEGs (Figure 1C). The loop graph illustrated the chromosomal positions of the 20 DE-CM-RGs (Figure 1D). Chromosomal localisation showed that four of the 20 DE-CM-RGs were located on chromosome 8, two genes were located on chromosomes 5, 10, 11 and 16, respectively, and there was only one gene on each of chromosomes 1, 4, 6, 12, 14, 15, 19 and the Y chromosome. Subsequently, 20 DE-CM-RGs were analyzed using GO and KEGG to identify their underlying functions. The DE-CM-RGs were functionally enriched in cholesterol metabolic process, cholesterol biosynthetic process, sterol biosynthetic process, and fatty acid synthase activity based on GO annotations for cellular components, biological processes, and molecular functions. Figure 2A displayed the top ten GO terms for each classification. The KEGG pathways enriched by these DE-CM-RGs included glycerolipid metabolism, cholesterol metabolism, fatty acid metabolism, PPAR signaling pathway, biosynthesis of unsaturated fatty acids, and steroid biosynthesis pathways (Figure 2B). The correlation among the twenty DE-CM-RGs was strong, with SQLE and HMGCS1 showing the highest level of strength, as evidenced by a robust correlation coefficient of 0.9508 (Figure 2C). The high correlation between SQLE and HMGCS1 reveals a tight synergistic regulation of the cholesterol synthesis pathway in specific cells or tissues. As key enzymes of this pathway, changes in their expression may affect hepatic cholesterol homeostasis and thus have a role in the progression of NAFLD.

A PPI network of 20 DE-CM-RGs was constructed, and a significant gene cluster module was identified, including 9 potential hub genes: MVK, FDPS, MVD, TM7SF2, FDFT1, SQLE, CYP7A1, HMGCS1, and NSDHL (Figures 2D,E).

### 3.2 MVK and TM7SF2 were protective factors, HMGCS1 and FDPS were risk factors for NAFLD

**FIGURE 3:**
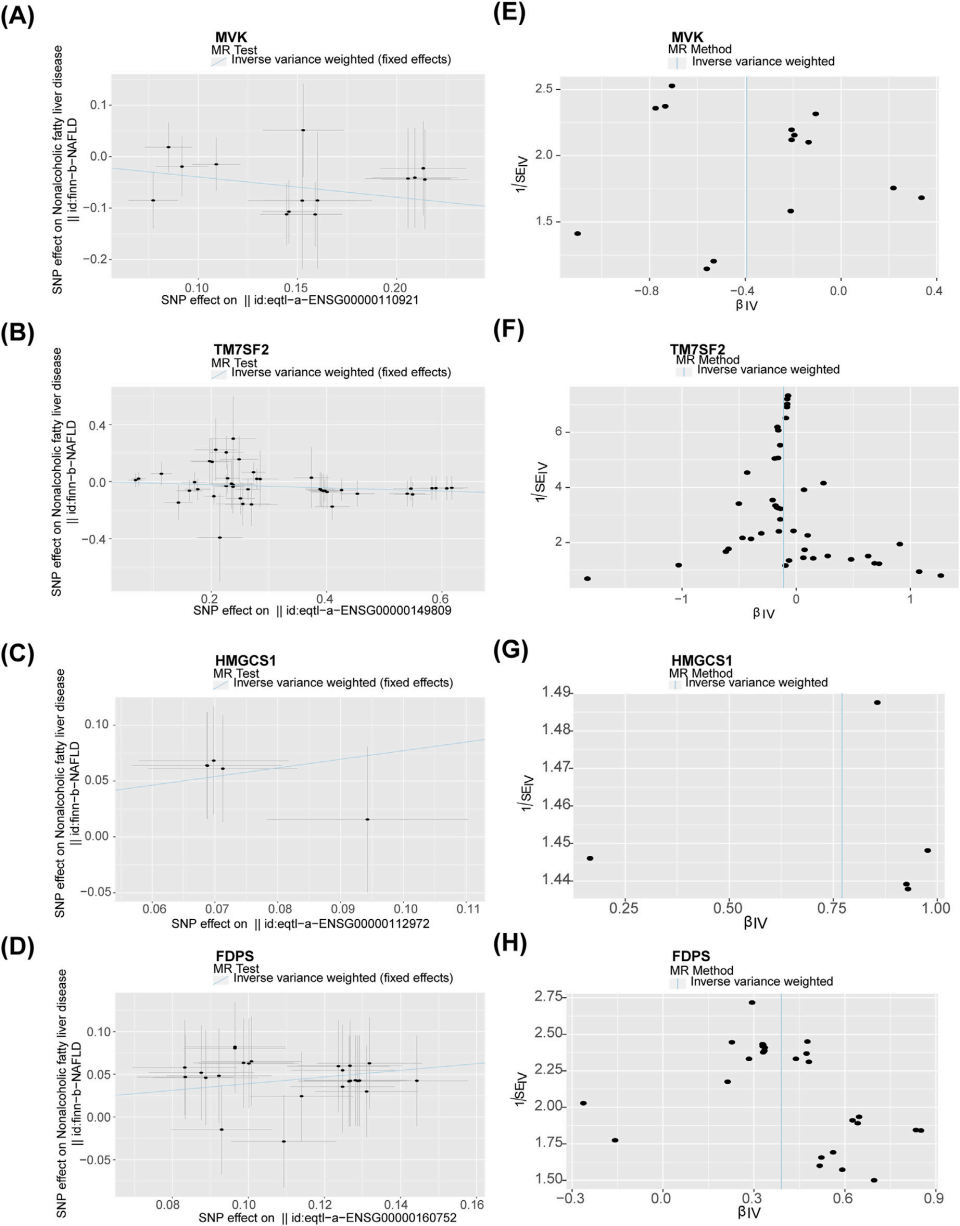
**(A–D)** Association of MVK, TM7SF2, HMGCS1, and FDPS with NAFLD visualized by scatter plots. **(E–H)** Funnel plots of the association of MVK,TM7SF2, HMGCS1, and FDPS with NAFLD. NAFLD, non-alcoholic fatty liver disease.

**FIGURE 4:**
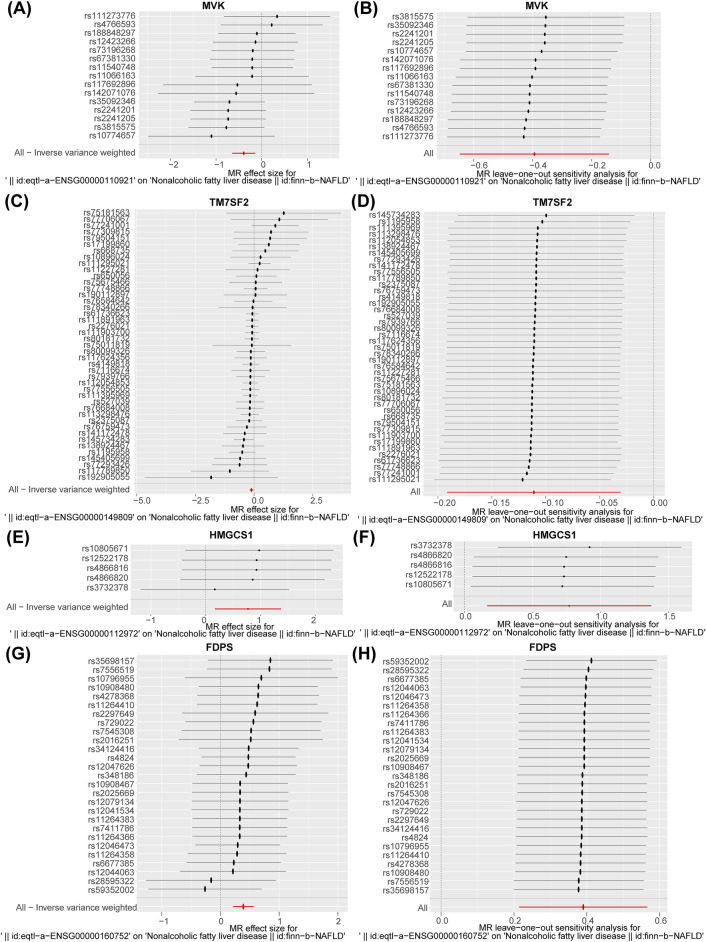
**(A, C, E, and G)** Forest plot of the association of MVK, TM7SF2, HMGCS1, and FDPS with NAFLD. **(B, D, F, and H)** Leave-one-out analysis of the causal association of MVK, TM7SF2, HMGCS1, and FDPS with NAFLD. The black dots and bars indicated the causal estimate and 95% CI when a SNP was removed in turn. The red dot and bar indicated the overall estimate and 95% CI using inverse variance weighted method. NAFLD, non-alcoholic fatty liver disease; CI, confidence interval; SNP, single-nucleotide polymorphism.

Nine potential hub genes were used as exposure factors and NAFLD as an outcome variable to explore the causal relationship between genes and NAFLD. Since there were too few SNPs corresponding to CYP7A1 and NSDHL to support the following analyses, both were excluded. The IVW found no evidence linking FDFT1, SQLE, and MVD to NAFLD. Of note, The IVW analysis provided suggestive evidence for the association between MVK (OR = 0.674, 95% CI = 0.524–0.868, *p* = 0.002), HMGCS1 (OR = 2.163, 95% CI = 1.182–3.955, *p* = 0.012), TM7SF2 (OR = 0.895, 95% CI = 0.826–0.970, *p* = 0.007), and FDPS (OR = 1.477, 95% CI = 1.239–1.761, *p* < 0.001) and the risk of NAFLD (Table 1). Specifically, MVK and TM7SF2 were protective factors, while HMGCS1 and FDPS were risk factors for NAFLD. These findings were visually represented using scatter plots, where the slopes of MVK and TM7SF2 were negative, while the slopes of HMGCS1 and FDPS were positive (Figures 3A–D). The approximately symmetric distribution of points in the funnel plots showed that the causality between MVK, TM7SF2, HMGCS1, FDPS and NAFLD followed Mendel’s second law of randomisation (Figures 3E–H). Forest plots illustrating the association between MVK,TM7SF2, HMGCS1 and FDPS with NAFLD are presented in Figures 4A,C,E,G respectively. The MR effect sizes for HMGCS1 and FDPS on NAFLD exceeded 0 in the forest plots, manifesting that they might increase the risk of NAFLD. However, the outcomes of MVK and TM7SF2 on NAFLD were opposite. The Cochran’s Q test did not detect any heterogeneity (Q_*p* > 0.05) (Supplementary Table S3). The MR-Egger regression analysis showed no significant overall horizontal pleiotropy based on the intercept value (*p* > 0.05) (Supplementary Table S3). The LOO analysis indicated that the overall estimate was not influenced by any individual SNP (Figures 4B,D,F,H). In conclusion, sensitivity analyses proved the reliability and robustness of MR outcomes. FDPS, HMGCS1, MVK, TM7SF2 identified as hub genes in this study by MR analysis.

**TABLE 1:** Associations of DE-CM-RGs with NAFLD.

	No. of SNPs	*p*	OR (95% CI)
FDFT1	49	0.548	1.014 (0.969–1.062)
SQLE	23	0.078	0.834 (0.682–1.021)
MVK	15	0.002	0.674 (0.524–0.868)
HMGCS1	5	0.012	2.163 (1.182–3.955)
TM7SF2	44	0.007	0.895 (0.826–0.970)
FDPS	27	0.000	1.477 (1.239–1.761)
MVD	10	0.963	0.993 (0.747–1.322)

DE-CM-RGs, the differentially expressed genes associated with cholesterol metabolism; NAFLD, non-alcoholic fatty liver disease; SNP, single-nucleotide polymorphism; CI, confidence interval; OR, odds ratio.

### 3.3 The hub gene-based nomogram could assess NAFLD risk accurately

**FIGURE 5:**
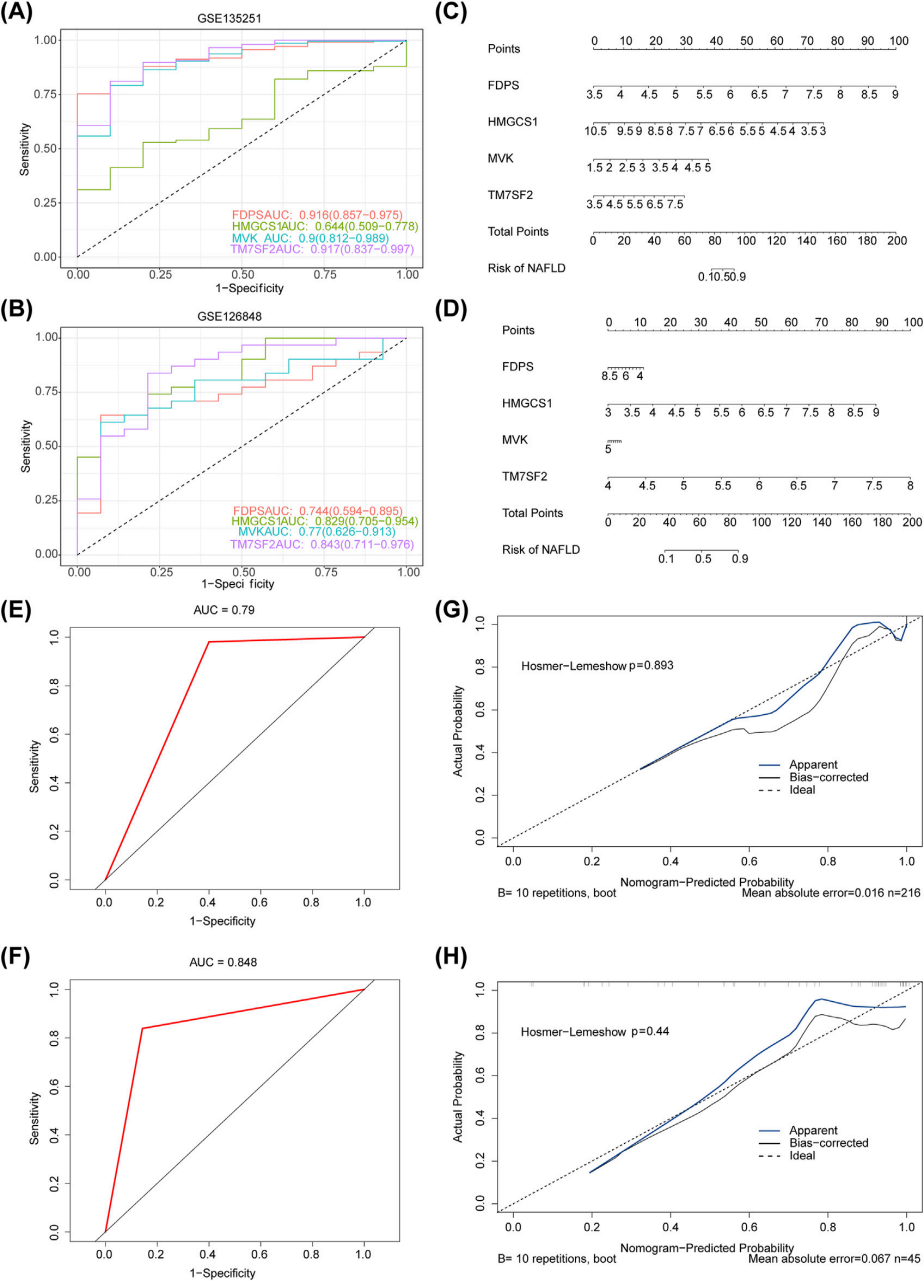
**(A,B)** ROC analysis of four hub genes (MVK, HMGCS1, TM7SF2 and FDPS) for diagnostic performance in GSE135251 and GSE126848 datasets. **(C,D)** Diagnostic nomogram for NAFLD using four hub genes (MVK, HMGCS1, TM7SF2 and FDPS) in the GSE135251 and GSE126848 datasets. **(E,F)** ROC curve of nomogram in GSE135251 and GSE126848. **(G,H)** Calibration curves of the nomogram in GSE135251 and GSE126848. ROC, receiver operating characteristic; AUC, area under the curve; NAFLD, non-alcoholic fatty liver disease.

The diagnostic accuracy of MVK, HMGCS1, TM7SF2, and FDPS for NAFLD was confirmed by ROC analysis, as the AUC values for FDPS, MVK, and TM7SF2 genes exceeded 0.7 in the datasets GSE135251 and GSE126848 datasets (Figures 5A,B). Nomograms containing these four hub genes were constructed based on GSE135251 and GSE126848 datasets (Figures 5C,D). The AUC of the nomogram was 0.79 in GSE135251 and 0.848 in GSE126848 (Figures 5E,F), demonstrating its strong predictive ability for NAFLD. The calibration curve demonstrated excellent concordance between the predictions derived from the nomogram and the actual observations (Figures 5G,H).

### 3.4 The latent functions of hub genes

**FIGURE 6:**
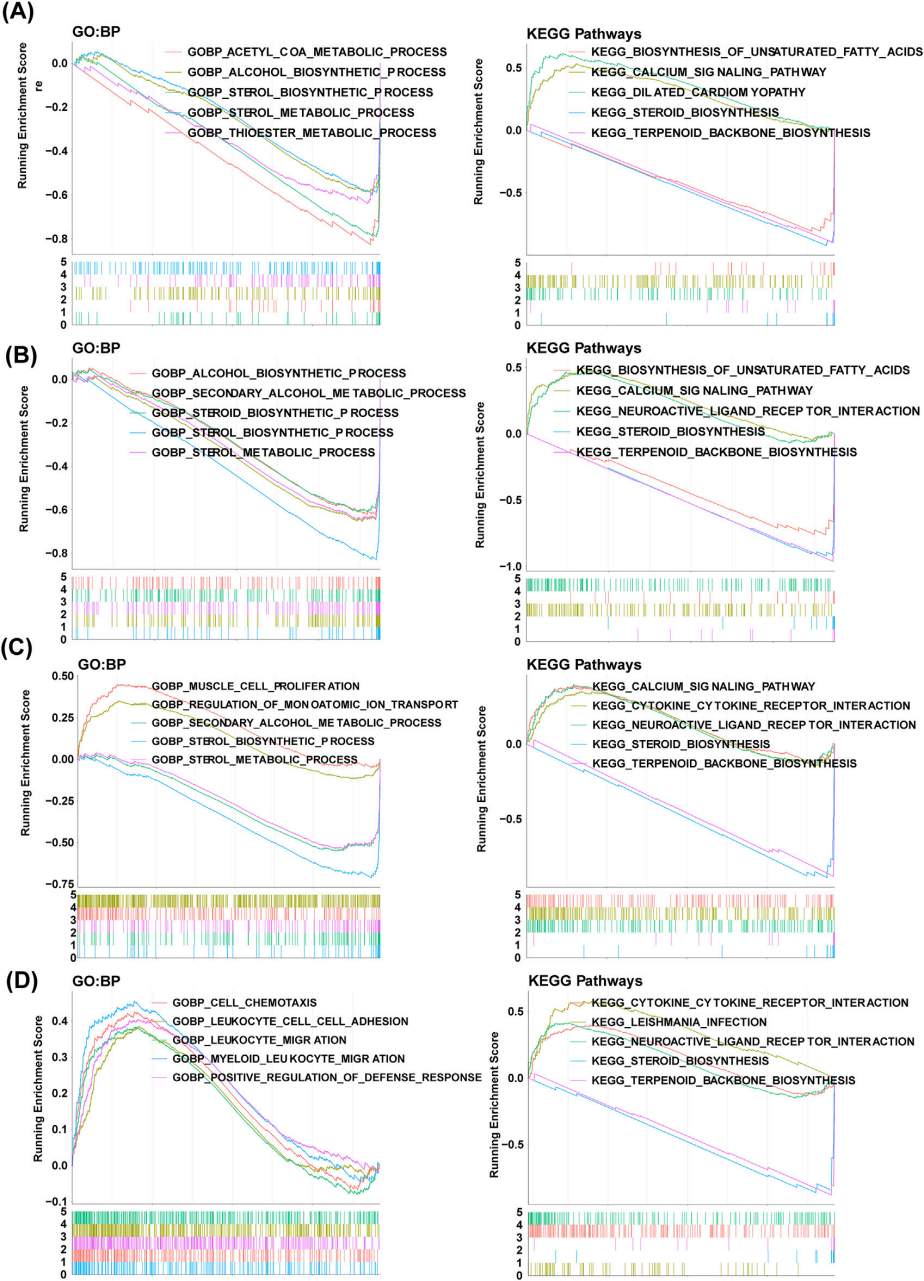
GSEA revealing GO-BP and KEGG items enriched by hub genes. The GSEA results of HMGCS1 **(A)**, FDPS **(B)**, MVK **(C)** and **(D)** TM7SF2. GO, Gene ontology; KEGG, Kyoto Encyclopedia of Genes and Genomes; BP, biological progress; GSEA, gene set enrichment analysis.

We conducted GSEA for the identification of key signaling pathways at a single-gene level. HMGCS1 was significantly enriched in thioester and sterol metabolic processes, steroid biosynthesis, calcium signaling pathway, and unsaturated fatty acid biosynthesis (Figure 6A). FDPS showed significant enrichment in secondary alcohol metabolism, steroid biosynthesis, calcium signaling pathway, and biosynthesis of unsaturated fatty acids (Figure 6B). MVK exhibited significant enrichment in secondary alcohol metabolism, sterol metabolic processes, regulation of monoatomic ion transport, steroid biosynthesis, and calcium signaling pathways (Figure 6C). TM7SF2 demonstrated significant enrichment in myeloid leukocyte migration functions, cell chemotaxis, leukocyte cell adhesion, leukocyte migration, and steroid biosynthesis (Figure 6D).

### 3.5 There were 8 differential immune cells in NAFLD and controls

**FIGURE 7:**
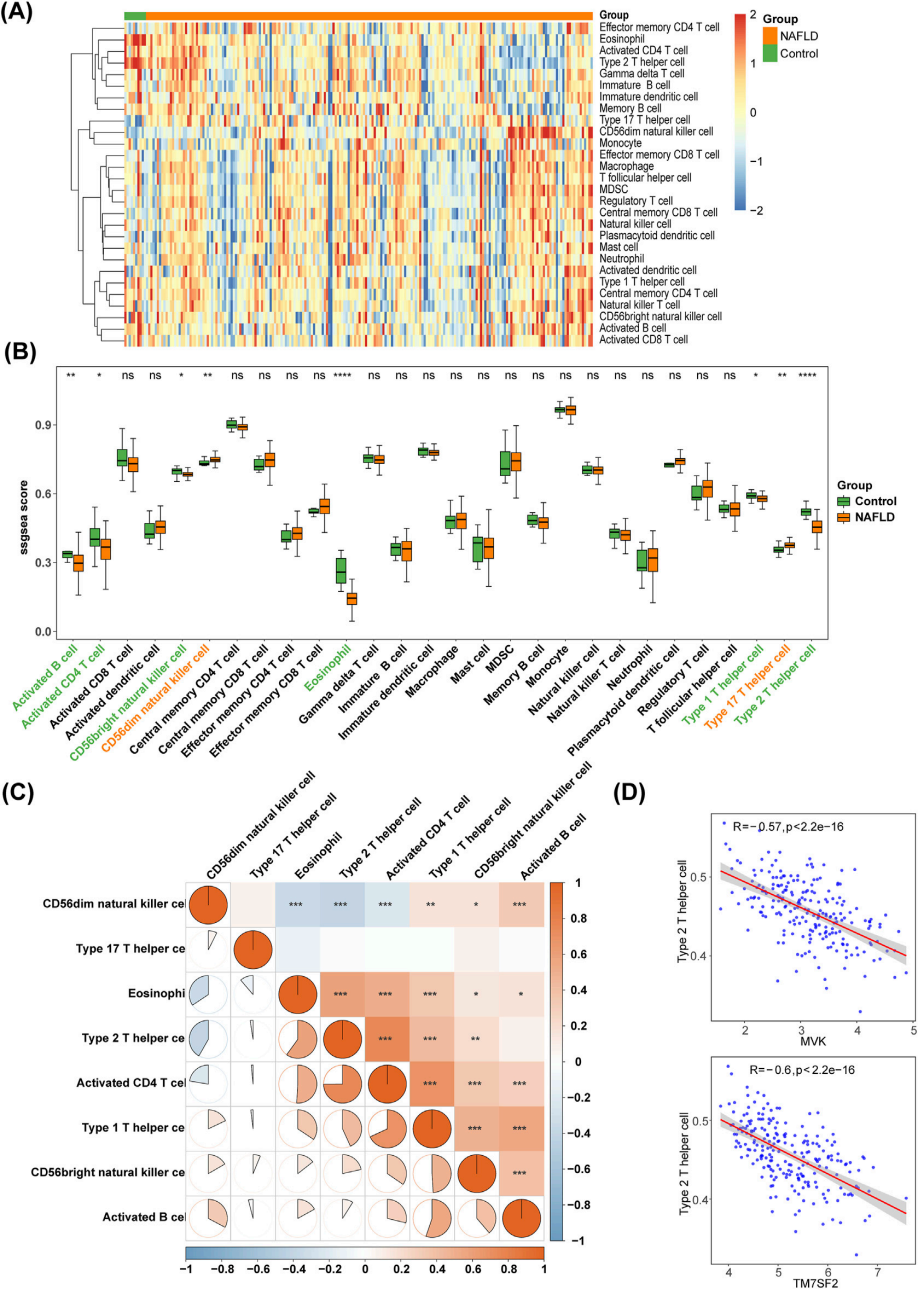
Immune infiltration analysis. **(A)** The heat map showing the distribution of 28 immune cells in NAFLD and controls. **(B)** Box plots comparing differences in enrichment scores of 28 immune infiltrating cells between NAFLD and controls. *p < 0.05, **p < 0.01, ***p < 0.001, ns: *p* > 0.05. **(C)** Heat map displaying correlations among different immune cells. **(D)** Scatter plot of correlation between hub genes (MVK and TM7SF2) and differential immune cells in NAFLD. NAFLD, non-alcoholic fatty liver disease.

The ssGSEA algorithm was used to analyze the enrichment scores of 28 immune cell types, and a heat map was generated to visualize immune infiltration in NAFLD (Figure 7A). Wilcoxon test revealed significant differences in activated CD4 T cells, CD56^bright^ natural killer (NK) cells, T helper Type 1 (Th1) cells, activated B cells, CD56^dim^ NK cells, Th17 cells, eosinophils, and Th2 cells between NAFLD and control groups (*p* < 0.05). Compared to controls, NAFLD patients had higher levels of CD56^dim^ NK cells and Th17 cells but lower levels of activated CD4 T cells, CD56^bright^ NK cells, Th1 cells, activated B cells, eosinophils and Th2 cells (Figure 7B). Th2 cells and activated CD4 T cells exhibited the strongest correlation with a coefficient of 0.7492 (Figure 7C). The correlation between the four hub genes (FDPS, HMGCS1, MVK, and TM7SF2) and eight differential immune infiltrating cells was analyzed, a negative association was observed between Th2 cells and both MVK and TM7SF2 (Figure 7D).

### 3.6 Comprehensive regulatory network analysis revealed key regulatory factors and potential therapeutic agents of hub genes

**FIGURE 8:**
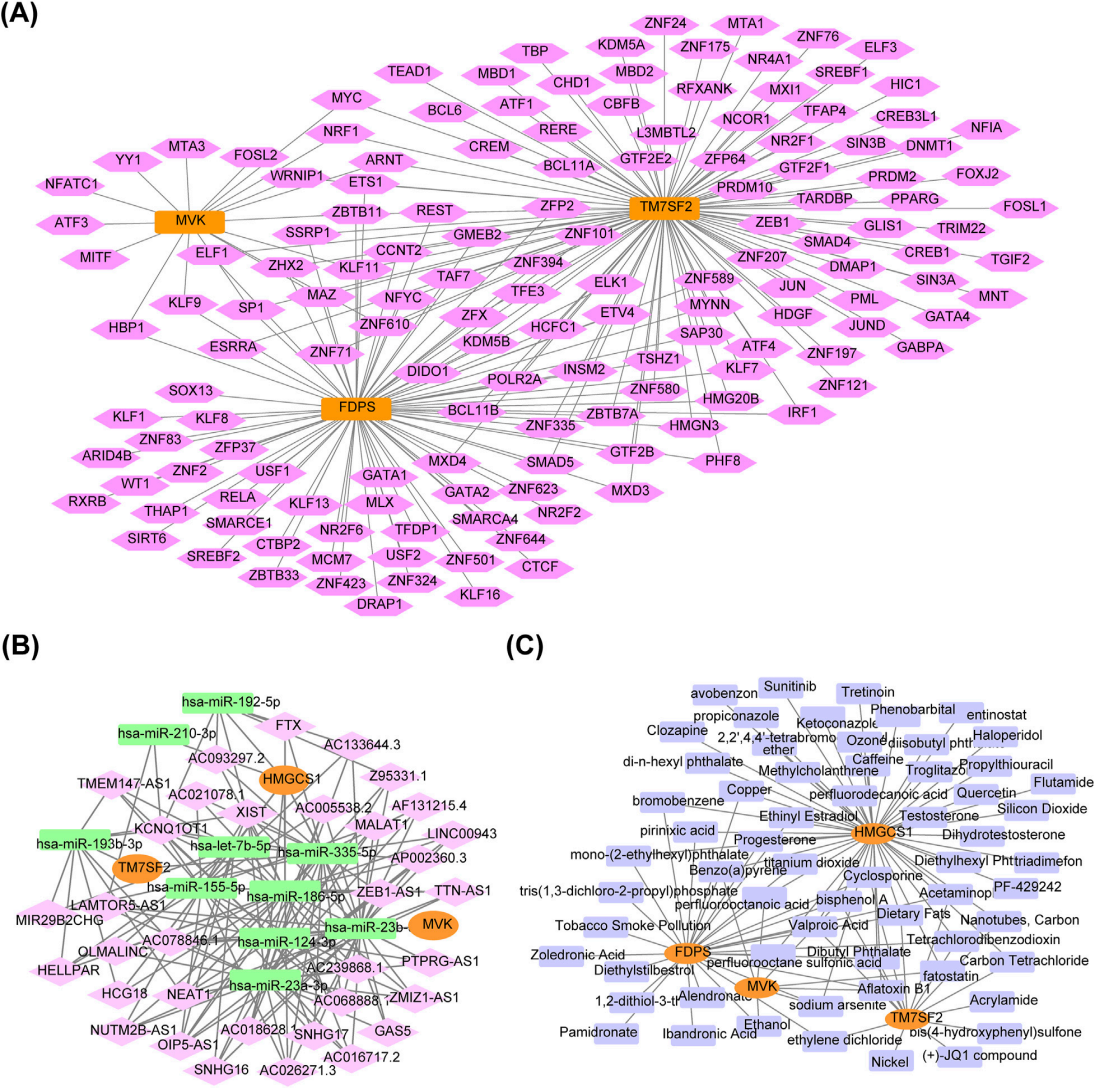
Regulatory network of hub genes. **(A)** mRNA-TF regulatory network. Orange for hub genes, pink for TFs. **(B)** lncRNA-miRNA-mRNA regulatory network. Orange represents hub genes, green represents miRNAs, pink represents lncRNAs. **(C)** mRNA-drug regulatory network. Orange for hub genes, purple for drugs. TFs, transcription factors; mRNA, messenger RNA; lncRNA, long non-coding RNA; miRNA, microRNA.

The mRNA-TF regulatory network included 153 nodes (3 hub genes, 150 TFs), and 206 edges (Figure 8A). The lncRNA-miRNA-mRNA regulatory network consisted of three genes, 47 nodes and 158 edges (Figure 8B). The findings revealed that the manifestation of HMGCS1 might be regulated in a competitive manner by fastening with hsa-let-7b-5p, hsa-miR-155-5p, hsa-miR-186-5p, hsa-miR-192-5p, hsa-miR-23a-3p, hsa-miR-210-3p, hsa-miR-335-p and hsa-miR-23b-3p to 134 lncRNAs. 24 lncRNAs could competitively bind both hsa-miR-335-5p and hsa-miR-193b-3p to regulate TM7SF2. Thirteen lncRNAs competitively bind to hsa-miR-124-3p, regulating the expression of MVK. The mRNA-drug regulatory network identified four key hub genes and 62 drugs or compounds, including tretinoin, entinostat, alendronate, zoledronic acid and quercetin as potential targeted drugs for regulating cholesterol metabolism genes in NAFLD (Figure 8C).

## 4 Discussion

NAFLD is a prevalent hepatic disorder affecting millions of individuals worldwide. NAFLD not only affects patients’ wellbeing and health, but also increases the risk of developing cirrhosis and HCC. Consequently, effective prevention and control strategies are imperative for curtailing the onset and progression of this condition. However, the current therapeutic approaches for NAFLD remain unsatisfactory due to the intricate nature of its pathogenesis. A previous study demonstrated that hepatic accumulation of free cholesterol induces cytotoxicity, thereby facilitating the transition from steatosis to non-alcoholic steatohepatitis (NASH) (Van Rooyen et al., 2011). The exact role of cholesterol metabolism in NAFLD is not fully understood. To our understanding, this research is the initial attempt to recognize and examine the involvement of CM-RGs in NAFLD.

In the present investigation, we demonstrated the significant impact of four genes (MVK, HMGCS1, TM7SF2, and FDPS) on NAFLD risk. Both datasets showed AUC values exceeding 0.7 for FDPS, MVK, and TM7SF2. Additionally, we constructed a nomogram model incorporating MVK, HMGCS1, TM7SF2, and FDPS. The nomogram achieved AUCs of 0.79 in GSE135251 and 0.848 in GSE126848 datasets respectively, indicating its reliability as a biomarker for predicting NAFLD diagnosis.

Our study identified four key DE-CM-RGs (MVK, HMGCS1, TM7SF2, and FDPS) with the strongest association to NAFLD. FDPS, a key enzyme in the mevalonate pathway, produces farnesyl pyrophosphate and geranyl pyrophosphate, which are involved in cholesterol synthesis. The presence of FDPS in various neoplasms and its association with cardiovascular diseases has been previously reported (Seshacharyulu et al., 2019; Wang et al., 2021). Excessive FDPS expression leads to heightened disease severity in NASH by increasing farnesyl pyrophosphate levels, which enhance CD36 expression and accelerate the development of NASH through lipid accumulation, inflammation, and fibrosis (Liu et al., 2023). Based on our MR analysis and nomogram model, we identified FDPS as a risk factor for NAFLD. Our findings suggested that increased FDPS expression was associated with an elevated risk of NAFLD, consistent with previous research (Liu et al., 2023). HMGCS1 is a key enzyme in the cholesterol biosynthesis pathway. Ursolic acid inhibits HMGCS1 activity, reducing cholesterol-related metabolite production and may explaining its therapeutic effects against hyperlipidemia and atherosclerosis and related disorders (Ma et al., 2022). Additionally, dysregulation of mevalonate on the CSN6-HMGCS1-YAP1 axis has been found to specifically promote NAFLD-related liver cancer progression in HCC development (Li et al., 2024). However, the exact involvement of HMGCS1 in NAFLD remains unclear. The TM7SF2 gene encodes a pivotal enzyme involved in cholesterol biosynthesis and plays a critical role in diverse biological processes, encompassing liver regeneration (Bartoli et al., 2016) and regulation of inflammatory response (Gatticchi et al., 2015). The investigation revealed that a deficiency in TM7SF2 leads to delayed cell cycle progression and disrupted lipid metabolism during liver regeneration (Bartoli et al., 2016). Leonardo Gatticchi et al. have confirmed that the deletion of TM7SF2 disrupts adipogenesis in mouse embryonic fibroblasts by modulating early and late regulators, leading to decreased insulin sensitivity and upregulation of the anti-adipogenic factor matrix metalloproteinase 3 (Gatticchi et al., 2021). Dysregulation of TM7SF2 function may contribute to metabolic abnormalities and diseases, including dyslipidemia, insulin resistance and obesity. However, the role of TM7SF2 in NAFLD remains to be elucidated. The MVK gene encodes the enzyme mevalonate kinase, which plays a vital role in the early stages of cholesterol biosynthesis. Mutations in the MVK gene can lead to hyperimmunoglobulinemia D syndrome and mevalonic aciduria (Brennenstuhl et al., 2021; Haas and Hoffmann, 2006). The upregulation of MVK was observed in NAFLD patients in this study; however, further investigation is required to elucidate its involvement in disease mechanisms.

The activity of HMGCS1 can influence the rate of cholesterol biosynthesis in the liver. Ligustilide forms an irreversible bond with the Cys129 site of HMGCS1 through its metabolic intermediate, leading to a significant reduction in HMGCS1 enzyme activity and thus effectively ameliorating dyslipidemia induced by a high-fat diet in mice (Zhang et al., 2023). HMGCS1 also serves as a crucial molecular connection between obesity, inflammation, type 2 diabetes, and coronary artery calcification (Ding et al., 2015). Furthermore, TM7SF2, a novel factor, is implicated in the differentiation of fat cells and the development of adipose tissue along with metabolic wellbeing (Gatticchi et al., 2021). Depletion of TM7SF2 may lead to various unfavorable metabolic outcomes including weight gain, decreased insulin sensitivity, and reduced Akt kinase activity (Gatticchi et al., 2020), potentially affecting hepatic lipid metabolism homeostasis due to differences in its expression or function (Huang et al., 2021). Additionally, Mireia Junyent et al. suggested that genetic variations in the MVK gene influence levels of high-density lipoprotein cholesterol, potentially affecting blood lipid levels (Junyent et al., 2009). In nephrotic rats, the upregulation of the FDPS gene significantly promotes cholesterol biosynthesis, providing a crucial insight into the pathogenesis of hypercholesterolemia associated with nephrotic syndrome (Zhou et al., 2008). Our review indicates that the four hub genes are closely linked to cholesterol synthesis and lipid metabolism, playing pivotal roles in metabolic disorders like hyperlipidemia, insulin resistance, diabetes, and obesity. Considering the known correlation between these conditions and NAFLD progression (Rinella et al., 2024), it is plausible that these hub genes are strongly associated with NAFLD progression.

Immune dysregulation plays an essential part in NAFLD pathogenesis. We studied immune infiltration in NAFLD and found elevated levels of CD56^dim^ NK cells and Th17 cells compared to control liver samples. Th17 cells significantly impact immune defense against pathogens that exist outside of cells. The population of Th17 cells is increased in both the hepatic and peripheral blood of NAFLD mice (He et al., 2017). Similarly, patients with NASH exhibit increased hepatic TH17 cell population (Rau et al., 2016). NK cells are classified into CD56^dim^ and CD56^bright^ types based on surface density, with the former exerting cytotoxicity and the latter regulating immunity through cytokine secretion (Stabile et al., 2017). Previous research has shown a decrease in CD56^bright^ NK cells in individuals with NAFLD compared to the control group, while an elevated frequency of CD56^dim^ NK cells has been observed, consistent with our own findings (Diedrich et al., 2020). In our investigation, Th2 cell levels decreased in NAFLD and were associated with MVK and TM7SF2, but their role in the pathogenesis of NAFLD remains unclear. Th2 cells can have anti-inflammatory effects through cytokine secretion. The frequency of Th2 cells was elevated in peripheral blood of individuals with NAFLD compared to controls (Rau et al., 2016), but no statistically significant disparities were noted between NASH patients and both NAFLD individuals and controls (Inzaugarat et al., 2011). IL-33 stimulates Th2 cells to produce cytokines, inducing liver fibrosis while reducing liver damage in a murine model of NASH (Gao et al., 2016). Hence, additional research is required to elucidate the precise involvement of Th2 cells in NAFLD.

GSEA showed that FDPS, HMGCS1 and MVK were significantly enriched in calcium signaling pathways. The dysregulation of calcium signaling is crucial in metabolism and has been linked to cancer development (Liang et al., 2018). RYR1 gene mutations are frequently found in both mouse and human NASH-HCC, indicating dysregulation of calcium signaling in cholesterol-related NASH-HCC and NASH rather than steatosis (Liang et al., 2018). PAR2 impairs glucose uptake and insulin sensitivity in NAFLD by decreasing GLUT2 expression through the Gq-MAPK-FoxA3 pathway, and inhibiting insulin-Akt signaling via the Gq-calcium-CaMKK2 pathways (Shearer et al., 2022). Unsaturated fatty acid involves FDPS and HMGCS1. The unsaturated fatty acids can inhibit lipolysis and mitigate hepatic fat accumulation (Rosqvist et al., 2019). Musa-Veloso et al. discovered that supplementation with ω3-polyunsaturated fatty acids effectively reduced liver fat content and steatosis score in NAFLD patients (Musa-Veloso et al., 2018). The previous study proposed that n-3 polyunsaturated fatty acids have the potential to modulate molecular pathways related to lipogenesis, endoplasmic reticulum function, and mitochondrial function, leading to improvements in NASH (Okada et al., 2018).

Considering the limited availability of effective treatments for NAFLD, this study explored gene-targeted medications that specifically focus on four hub genes. Tretinoin is a vitamin A-derived retinoid drug commonly used to treat acne and acute promyelocytic leukemia. Individuals with NAFLD exhibit decreased levels of circulating retinoic acid, which plays a crucial role in hepatic lipid metabolism and insulin resistance (Liu et al., 2015). Retinoic acid protected against high-fat diet-induced hepatosteatosis by downregulating Srebp-1c expression and enhancing antioxidant capacity through a Sirt1-mediated mechanism (Geng et al., 2017). Alendronate, a pharmacological inhibitor of FDPS used clinically to treat glucocorticoid-induced osteoporosis, improved inflammation, steatosis and fibrosis in mice with NASH(27). Zoledronic acid, an inhibitor of FDPS, mitigated hepatic steatosis by suppressing *de novo* lipogenesis in NAFLD mice (Mohamed et al., 2019). Quercetin ameliorates NAFLD in db/db mice by attenuating inflammation, oxidative stress, and modulating lipid metabolism via the farnesoid X receptor 1/Takeda G-protein-coupled receptor 5 signaling pathways (Yang et al., 2019). Pharmaceutical interventions targeting these four genes present a novel prospective strategy for managing NAFLD.

However, our study has several limitations. First, the limited availability of information in the public datasets necessitates the inclusion of additional data to increase the validity of the findings. Second, further validation through mouse experiments and additional clinical samples is imperative to corroborate the findings and elucidate the underlying mechanisms of CM-RGs in NAFLD.

## 5 Conclusion

In the current study, we have successfully identified four hub genes (MVK, HMGCS1, TM7SF2, and FDPS) associated with cholesterol metabolism in NAFLD. Based on these hub genes, we developed a nomogram to accurately diagnose patients with NAFLD. Our findings offer potential molecular targets for elucidating the pathogenesis of NAFLD and guiding the development of drug therapies. However, the specific mechanisms involved in disease development and the exact molecular targets require additional verification.

## Data Availability

Publicly available datasets were analyzed in this study. This data can be found here: The datasets supporting the conclusions of this article are available in the GEO repository, unique persistent identifier and hyperlink to dataset(s) in https://www.ncbi.nlm.nih.gov/gds/?term=GSE135251 and https://www.ncbi.nlm.nih.gov/gds/?term=GSE126848.
