# Supplementary material for: Identification of novel targets associated with cholesterol metabolism in nonalcoholic fatty liver disease: a comprehensive study using Mendelian randomization combined with transcriptome analysis
Source: Front Genet. 2024 Sep 18;15:1464865. doi: 10.3389/fgene.2024.1464865 (PMC11445148; doi:10.3389/fgene.2024.1464865)
Supplement: Supplementary file 1 [file Table1.DOCX]

Supplementary Table 1: Baseline characteristics of the GSE135251 and GSE126848 datasets

| Group | | | n | men | Age, yr | BMI, kg/m2 |
| --- | --- | --- | --- | --- | --- | --- |
| GSE126848 | NAFLD group | NAFL | 15 | 9 | 39.4 ± 10.6 | 32.8 ± 5.1 |
|  |  | NASH | 16 | 12 | 38.9 ± 17.0 | 33.9 ± 6/2 |
|  | Control group | | 14 | 14 | 39.5 ±12.0 | 23.1 ± 1.6 |
| GSE135251 | NAFLD group | | 206 | 123 | 54 ± 11.87 | 31.34 ± 5.04 |
|  | Control group | | 10 | NA | NA | NA |

NAFL, nonalcoholic fatty liver; NASH, nonalcoholic steatohepatitis; NAFLD, non-alcoholic fatty liver disease.

Supplementary Table 2: SNP information for the hub gene

| Gene | No.of SNPs | Reference SNP ID |
| --- | --- | --- |
| MVK | 15 | rs4766593, rs142071076, rs117692896, rs11540748,  rs2241201, rs12423266, rs10774657, rs35092346,  rs111273776, rs11066163, rs188848297, rs73196268,  rs3815575, rs2241205, rs67381330 |
| HMGCS1 | 5 | rs3732378, rs4866816, rs4866820, rs10805671, rs12522178 |
| TM7SF2 | 44 | rs138924467, rs668735, rs77556505, rs117789850,  rs77293426, rs145405699, rs77309815, rs11227281  rs4149818, rs75011819, rs111395969, rs2276021  rs75675466, rs76759473, rs113298476, rs117624356,  rs75181563, rs77241001, rs112054853, rs650056  rs111295021, rs77748866, rs76584642, rs79504151  rs2375087, rs192905055, rs527039, rs7939766  rs111903700, rs1195958, rs190112897, rs77731681  rs145734283, rs78340266, rs77706067, rs17199860  rs76684008, rs111891963, rs61736623, rs80099326  rs7116674, rs80181732, rs10896024, rs141172478 |
| FDPS | 27 | rs12046473, rs11264358, rs11264366, rs348186  rs729022, rs4278368, rs2016251, rs28595322  rs7545308, rs2297649, rs4824, rs35698157  rs10908467, rs7556519, rs11264383, rs59352002  rs6677385, rs2025669, rs12041534, rs12079134  rs12047626, rs11264410, rs10796955, rs34124416  rs10908480, rs12044063, rs7411786 |

SNP, single-nucleotide polymorphism.

Supplementary Table 3: Sensitive analysis for Mendelian randomization analysis

| Gene | Method | Heterogeneity | | Pleiotropy | | |
| --- | --- | --- | --- | --- | --- | --- |
|  |  | Q | Q_pvalue | Egger  intercept | Intercept’s se | P-value |
| MVK | Inverse variance weighted | 7.796 | 0.900 | -0.011 | 0.058 | 0.856 |
|  | MR Egger | 7.762 | 0.859 |  |  |  |
| HMGCS1 | Inverse variance weighted | 0.974 | 0.914 | 0.200 | 0.204 | 0.399 |
|  | MR Egger | 0.012 | 1.000 |  |  |  |
| TM7SF2 | Inverse variance weighted | 23.922 | 0.989 | 0.015 | 0.027 | 0.589 |
|  | MR Egger | 23.622 | 0.986 |  |  |  |
| FDPS | Inverse variance weighted | 5.966 | 1.000 | 0.060 | 0.065 | 0.367 |
|  | MR Egger | 5.121 | 1.000 |  |  |  |
